# Development and Characterisation of the Imiquimod Poly(2-(2-methoxyethoxy)ethyl Methacrylate) Hydrogel Dressing for Keloid Therapy

**DOI:** 10.3390/polym9110579

**Published:** 2017-11-05

**Authors:** Wei-Chih Lin, Sin-Han Liou, Yohei Kotsuchibashi

**Affiliations:** 1 Department of Mechanical and Electromechanical Engineering, National Sun Yat-sen University, Kaohsiung 80424, Taiwan; polo14772g@gmail.com; 2 Department of Materials and Life Science, Shizuoka Institute of Science and Technology, Shizuoka 437-8555, Japan; kotsuchibashi.yohei@sist.ac.jp

**Keywords:** MEO_2_MA, imiquimod, keloid fibroblast

## Abstract

The imiquimod-poly(2-(2-methoxyethoxy)ethyl methacrylate) hydrogel (poly(MEO_2_MA) hydrogel) dressing was developed for the keloid therapy application. Four groups of the hydrogels, including the imiquimod-poly(MEO_2_MA) hydrogel, crosslinked with 0.2 mol %, 0.4 mol %, 0.6 mol %, and 0.8 mol % of di(ethylene glycol) dimethacrylate cross-linker (DEGDMA), were synthesised and characterised for fabricating the imiquimod-poly(MEO_2_MA) hydrogel pad. The lower critical solution temperature (LCST) of the poly(MEO_2_MA) hydrogel was measured at approximately 28 °C and was used as a trigger to control the imiquimod loading and release. The loaded amounts of the imiquimod in the poly(MEO_2_MA) hydrogel, crosslinked with 0.2 mol % and 0.8 mol % of DEGDMA, were about 27.4 μg and 14.1 μg per 1 mm^3^ of the hydrogel, respectively. The imiquimod-release profiles of two samples were characterised in a phosphate buffered saline (PBS) solution at 37 °C and the released imiquimod amount were about 45% and 46% of the total loaded imiquimod. The Cell Counting Kit-8 (CCK-8) assay was utilised to analyse the cell viability of keloid fibroblasts cultured on the samples of imiquimod-poly(MEO_2_MA) hydrogel, crosslinked with 0.2 mol % and 0.8 mol % of DEGDMA. There was around a 34% decrease of the cell viabilities after 2 days, compared with the pure-poly(MEO_2_MA) hydrogel samples. Therefore, the developed imiquimod-poly(MEO_2_MA) hydrogel dressing can affect the proliferation of keloid fibroblasts. It should be possible to utilise the hydrogel dressing for the keloid therapy application.

## 1. Introduction

Wounds are typically referred to as the damage of tissue or outer skin injuries caused by physical means. Wound healing is a process of regenerating damaged or lost cellular tissue layers and skin [1,2,3,4]. Pathological scars, such as hypertrophic scars and keloid scars, are one of the potential results of the wound healing process. In the healing process, keloid formation is related to the over-proliferation of keloid fibroblasts causes by the alterations of the extracellular matrix (ECM) [4,5]. Keloid scars with a high recurrence rate grow beyond the boundaries of the original wound and are sometimes accompanied by pain and itchiness [6,7]. Therapeutic treatments, such as removal surgery, cortisone injection, cryotherapy, irradiation, and laser treatment, have been used to remove keloids in clinical applications [8]. The 5% imiquimod cream is the most common and widely used in clinical practice, due to several advantages, such as the fact it is simple and quick to use and is low-cost. The imiquimod is an immune response modifier and stimulates interferon, a proinflammatory cytokine that can effectively make collagen collapse. It has been suggested as an effective way to reduce the recurrence of keloid [9]. 

The used amounts and concentrations of the imiquimod also play a critical role. For example, Berman and Kaufman discussed the effects of postoperative imiquimod 5% cream on 12 patients with keloid disease for 24 weeks [10]. The final assessment showed none of the keloids had recurred but a few patients experienced side effects, including mild hyperpigmentation, pruritus, and clinical impetiginisation. Therefore, a stable, reliable, and controllable treatment is needed to precisely release the requested amount of imiquimod to scars over a long period of time for the keloid therapy.

Polymeric materials have been frequently used in modern polymer wound dressings as temporary skin repair and protection substitutes, such as polymer membranes and bio-scaffolds [11,12,13,14,15,16,17]. Hydrogel, which is an insoluble hydrophilic polymer, has emerged as a promising material for wound dressing. It provides high-water content (70–90%), which maintains a moist condition for the tissue-grown microenvironment [14,15,18,19,20]. The hydrogel is also a non-irritant, is non-reactive with biological tissue, and is permeable to the metabolites of wounds [19]. For instance, hydrogel was utilised as the main material for fabricating a stimulus-responsive, hydrogel-based drug-delivery system (DDS) [1,18,21].

The poly(2-(2-methoxyethoxy)ethyl methacrylate (poly(MEO_2_MA)) is an ethylene glycol-based polymer with a temperature-responsive property, which is known to show the reversible hydrophilic/hydrophobic property at a lower critical solution temperature (LCST) of around 28 °C [22,23]. Furthermore, poly(MEO_2_MA) hydrogel is an ethylene glycol-based material, which was widely utilised in various fields because of its biocompatibility [24,25]. For example, a research team demonstrated the release of doxorubicin from the amphiphilic (CS-g-PCL(-g-P(MEO_2_MA-co-OEGMA))) copolymers by altering the temperatures [26]. The drug releasing was based on the deswelling behaviour and can be controlled accurately by temperature changes.

**Figure 1:**
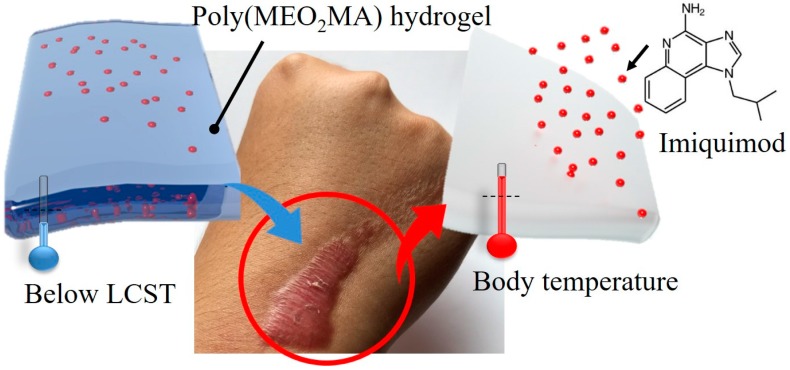
Schematic illustration of our purposed imiquimod-poly(MEO_2_MA) hydrogel-based treatment for keloid therapy. LCST: lower critical solution temperature.

In this research, we proposed a keloid therapy treatment based on the imiquimod-poly(MEO_2_MA) hydrogel dressing. The imiquimod is embedded into the poly(MEO_2_MA) hydrogel and can be released by changing the external temperatures, as shown in Figure 1. Poly(MEO_2_MA) hydrogels with four different molar feed ratios of the cross-linker were synthesised, and their fundamental properties, including the LCST and swelling/deswelling, were characterised by using ultraviolet-visible spectroscopy (UV-Vis), analytical balance, and enzyme-linked immunosorbent assay (ELISA). The imiquimod releasing profiles of the fabricated imiquimod-poly(MEO_2_MA) dressings were measured in phosphate buffered saline (PBS) solution at 37 °C. The keloid fibroblasts were cultured on the fabricated dressing, and Cell Counting Kit-8 (CCK-8) assay was applied to study the cell viability of the keloid fibroblasts.

## 2. Fabrication and Characterisation

The fabrication and characterisation of the poly(MEO_2_MA) hydrogel dressing play vital roles in this research. There are three steps in the fabrication procedure, which are the hydrogel synthesis, membrane formation, and drug loading process. The characterisations contain several parts including the studies of the poly(MEO_2_MA) hydrogel properties, the drug-releasing behaviour, and the performance of fabricated poly(MEO_2_MA) hydrogel pad. The details of the experiment settings and utilised chemicals and materials are described in the following sections.

### 2.1. Preparation of Poly(MEO_2_MA) Hydrogel Samples

**Figure 2:**
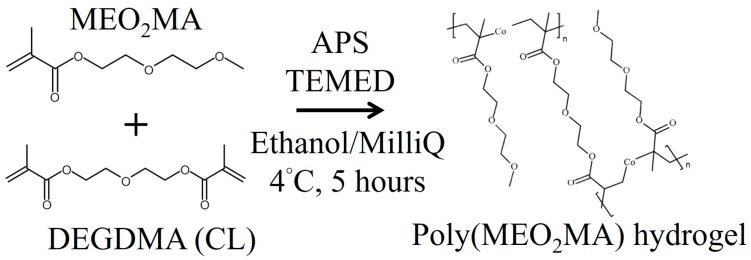
The synthesis of the poly(2-(2-methoxyethoxy)ethyl methacrylate) hydrogel (poly(MEO_2_MA) hydrogel) by redox polymerisation. DEGDMA (cross-linker; CL): di(ethylene glycol) dimethacrylate; APS: ammonium persulphate; TEMED: *N*,*N*,*N*′,*N*′-tetramethylethylenediamine.

The synthesis of the poly(2-(2-methoxyethoxy)ethyl methacrylate) hydrogel (poly(MEO_2_MA) hydrogel) is based on redox polymerisation, as presented in Figure 2. Chemicals used for the hydrogel synthesis were the MEO_2_MA monomer (Sigma-Aldrich, St. Louis, MO, USA, 95%), the di(ethylene glycol) dimethacrylate cross-linker (DEGDMA, Sigma-Aldrich, 95%), the ammonium persulphate initiator (APS, Wako Pure Chemical Industries, Tokyo, Japan), and the *N*,*N*,*N*′,*N*′-tetramethylethylenediamine (TEMED, Wako Pure Chemical Industries, Tokyo, Japan, 99%). The synthesis procedure started from the mixture of the DEGDMA cross-linker and the MEO_2_MA monomer. For making the poly(MEO_2_MA) hydrogel, crosslinked with 0.2 mol %, 0.4 mol %, 0.6 mol %, and 0.8 mol % of DEGDMA, and 10 mg, 20 mg, 30 mg, and 40 mg of the DEGDMA cross-linker solution, were mixed separately with 3760 mg of MEO_2_MA monomer and 10 mL of the water/ethanol (Vw:Ve ; 1:1) solution, [22,23,27]. Then, 40 mg of the APS powder was dissolved in 0.4 mL of water/ethanol solution and added into the monomer/cross-linker/water/ethanol solution. The final step was to add 31 mg of the TEMED to the hydrogel solution.

**Figure 3:**
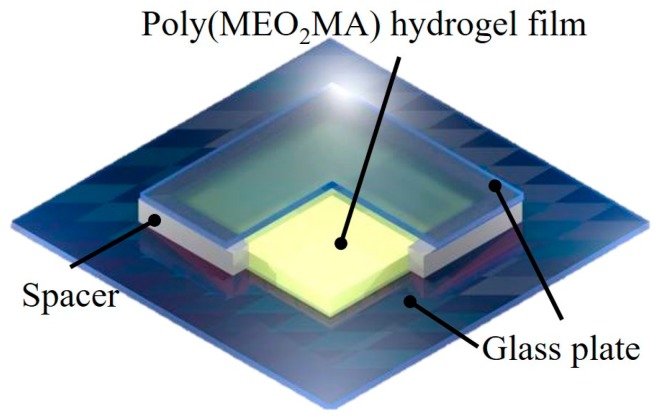
The illustration of the casting process to fabricate poly(MEO_2_MA) hydrogel film.

Subsequently, the poly(MEO_2_MA) hydrogel solution was filled in an enclosed space, formed by two glass plates with 1 mm thickness of the framework-spacer. The poly(MEO_2_MA) hydrogel films were fabricated using casting method, as shown in Figure 3. The casting mold, then, was kept in a refrigerator at 4 °C for redox polymerisation. After 5 h, the poly(MEO_2_MA) hydrogel membrane could be formed and removed from the casting mold. For the further experiments, a hole-puncher was utilised to make the standard disc hydrogel samples with a diameter of 6 mm from the hydrogel films. Later, the samples were immersed in Milli-Q water/ethanol mixture for 2 days to rinse the remaining chemicals, and the samples were then dried at room temperature until the weight constant of the hydrogel was achieved.

### 2.2. Preliminary Study of Poly(MEO_2_MA) Hydrogel Properties

Temperature-responsive poly(MEO_2_MA) hydrogel exhibits several unique properties, including transmittance, swelling, and deswelling behaviour, in response to the external temperature changes [28]. To utilise the swelling and deswelling properties for the drug loading and releasing purposes, the first step to utilise the swelling and deswelling properties for the drug loading and releasing is to measure the responsive temperature of the poly(MEO_2_MA) hydrogel, known as the LCST. The measuring details of the swelling and deswelling ratios are described in the section below.

#### 2.2.1. Lower Critical Solution Temperature of Poly(MEO_2_MA) Hydrogel

The transmittance transfers from the transparent state to the turbid and opaque state, while the external temperature alters starting from low temperature to the LCST. These behaviour changes are reversible and switchable and occur simultaneously. Therefore, the ultraviolet-visible spectroscopy (V-770, JASCO, Easton, MD, USA) was used to determine the LCST quantitatively where the measurement was based on the transmittance changes. The transmittance of poly(MEO_2_MA) hydrogel, crosslinked with 0.2 mol %, 0.4 mol %, 0.6 mol %, and 0.8 mol % of DEGDMA samples, was characterised individually from 4 °C to 37 °C by using the ultraviolet-visible spectrophotometer with the scan rate of 2 °C min^−1^ in Milli-Q water, dimethylformamide (DMF), and PBS solutions [29,30]. Before placing the samples on the quartz cuvette of the UV-Vis spectrophotometer, each sample was kept in Milli-Q water, DMF, and PBS solutions at 4 °C for 72 h to achieve the equilibrium swelling state.

#### 2.2.2. Characterisation of Swelling Property

The volume of the poly(MEO_2_MA) hydrogel performs the dynamic changes during the swelling and deswelling process. Equation (1) has been frequently applied to estimate the values of the swelling ratios by measuring the weight changes after the swelling. The swelling ratio, *Q_t_*, was calculated by the following Equation [18,19,25]:

(1) $$Q_{t}=\frac{\left(m_{t}-m_{d}\right)}{m_{d}}$$

where *m_t_* is the mass of swollen hydrogel at time t, and *m_d_* is the weight of the dried hydrogel.

Due to its better dissolving capability, the DMF (Sigma-Aldrich, 99%), an excellent hydrophilic aprotic solvent [31,32,33], was utilised to dissolve the imiquimod for the loading process. The first experimental step was to prepare the dried poly(MEO_2_MA) hydrogel, crosslinked with 0.2 mol %, 0.4 mol %, 0.6 mol %, and 0.8 mol % of DEGDMA samples, by placing it in a vacuum pumping system for 24 h. The dried samples, then, were weighted individually using analytical balance (Sartorius, Goettingen, Germany, BSA224S-CW Analytical Balance). Next, the dried samples were immersed separately in DMF, Milli-Q water, and PBS solutions for 72 h. The samples were removed respectively from the solution and wiped with tissue papers. The weighting process was repeated to measure the weight of the swollen poly(MEO_2_MA) hydrogel during the 72-hour period. Finally, the swelling ratio was calculated following the Equation (1).

#### 2.2.3. Characterisation of Deswelling Property

The swelling and deswelling responses of poly(MEO_2_MA) hydrogel are switchable and reversible. During the deswelling procedure, the volume of the poly(MEO_2_MA) hydrogel naturally shrinks to release the solution from the hydrogel. The same swelling measurement procedure was repeated to characterise the deswelling ratios of the poly(MEO_2_MA) hydrogel. Poly(MEO_2_MA) hydrogel, crosslinked with 0.2 mol %, 0.4 mol %, 0.6 mol %, and 0.8 mol % of DEGDMA samples, was operated individually in Milli-Q water and PBS solutions, where PBS is a solution with ion concentration close to the human blood plasma, at 4 °C for 72 h. Then, the samples were weighted at 10 °C, 20 °C, 25 °C, 30 °C, and 37 °C in each 3 h. Finally, the deswelling ratios were also calculated by Equation (1).

### 2.3. Fabrication of Imiquimod-Poly(MEO_2_MA) Hydrogel

The fabrication of imiquimod-poly(MEO_2_MA) hydrogel dressing contains two steps which are the synthesis and imiquimod loading processes. The poly(MEO_2_MA) hydrogel, crosslinked with 0.2 mol % of DEGDMA, performed better with regard to the swelling ratio, so it was selected for making the imiquimod-poly(MEO_2_MA) hydrogel dressing. In the imiquimod loading process, DMF was used as a drug carrier to load imiquimod into the poly(MEO_2_MA) hydrogel pad. The reason is that imiquimod is a kind of water-insoluble drug. Thus, the imiquimod loading using DMF into the poly(MEO_2_MA) hydrogel films with 0.2 mol % cross-linker was studied for the fabrication of imiquimod-poly(MEO_2_MA) dressing. Furthermore, the imiquimod-release kinematic in PBS solution was characterised to investigate the performance of the fabricated imiquimod-poly(MEO_2_MA) dressing, and it is presented in the part below.

#### 2.3.1. Imiquimod Loading

The imiquimod loading process starts from the preparation of the mixed imiquimod and DMF solution. One mg of the imiquimod (Tokyo Chemical Industry Co., Ltd, Tokyo, Japan, 98%) was added to 1 mL of DMF solution. Then, the disc poly(MEO_2_MA) hydrogel samples were immersed into the imiquimod/DMF solution at 25 °C for 12 h. The imiquimod can be loaded into the poly(MEO_2_MA) hydrogel film following the natural absorption during the swelling process. The samples, then, were removed out from the solution and were placed in the desiccator with a pumping system to exhaust DMF for 24 h. The last step was to immerse the dry disc imiquimod-poly(MEO_2_MA) hydrogel samples in the 0.46 mL of PBS solution, which was based on the result of measured swelling ratios, at 4 °C for 72 h. The equilibrium of imiquimod-poly(MEO_2_MA) hydrogel samples were covered by the aluminium foil and kept in the refrigerator for further experiments.

#### 2.3.2. Study of Imiquimod Releasing Profile

The release kinetics of the imiquimod in PBS solution was measured to evaluate the performance of the fabricated imiquimod-poly(MEO_2_MA) hydrogel pad. The procedures for testing the deswelling property of the poly(MEO_2_MA) hydrogel were repeated to characterise the imiquimod-release behaviours. The equilibrium-swollen hydrogel samples were kept in 100 mL of PBS solution at 37 °C to mimic the environment of human body temperature, at the time intervals. During the 120-h releasing period, 1 mL of PBS solution was extracted and measured using UV-Vis spectrometer (JASCO V-770) at λ = 244 nm; the imiquimod has a strong adsorption at 244 nm [34]. Besides, the same measuring procedure was applied to obtain the UV absorption values of 5 different concentrations of imiquimod/PBS solution to acquire the calibration curve. Finally, the released amount of imiquimod from the imiquimod-poly(MEO_2_MA) hydrogel dressing was obtained by comparing the UV absorption with the calibration curve result.

### 2.4. Performance Evaluation of Fabricated Imiquimod-Poly(MEO_2_MA) Hydrogel

The cell viability experiment was conducted to evaluate the performance of the fabricated imiquimod-poly(MEO_2_MA) hydrogel pad. The CCK-8 assay and the ELISA were applied to measure the optical density (OD) variations of the medium, which was used to culture the keloid fibroblasts. Then, the measured OD values were calculated to obtain the cell viability of keloid fibroblasts cultured on imiquimod-poly(MEO_2_MA) hydrogel dressing. The same procedures were also applied to measure the cell viability of the pure-poly(MEO_2_MA) hydrogel samples, as a control group to study the effect of released imiquimod to keloid fibroblasts. 

Before cell viability experiment, the imiquimod-poly(MEO_2_MA) hydrogel samples were sterilised using UV light irradiation and were placed in the 24 well-plates. The culture medium, which contains 90 mL of Dulbecco’s Modified of Eagle Medium (DMEM with 4.5 g/L glucose, l-glutamine, and sodium pyruvate, Corning Inc, Corning, New York, NY, USA.), 10 mL of foetal bovine serum (FBS, NQBB, Central, Hong Kong), 0.5 mL of penicillin/streptomycin (GEMINI, West Sacramento, CA, USA) and 0.5 mL of antibiotic/antimycotic (GEMINI, West Sacramento, CA, USA), was prepared and added into the well-plates. Keloid fibroblasts (Bioresource Collection and Research Center (BCRC), Hsinchu, Taiwan) were also prepared following the standard protocol from the BCRC. Then, keloid fibroblasts with a density of 3.0 × 10^4^ cells were added into the well-plates and cultured separately on the top surface of the pure-poly(MEO_2_MA) hydrogel sample and imiquimod-poly(MEO_2_MA) samples. The well-plates were stored in an incubator at 37 °C and 5% CO_2_ for 22 h. Continuously, a 0.01 mL of CCK-8 reagent was added to the culture plates and kept again in the incubator under the same conditions for 2 h. The final step was to extract 0.1 mL of culture medium, which was measured by using ELISA at 450 nm to study the cell viabilities of keloid fibroblasts.

## 3. Results and Discussion

### 3.1. Synthesis of Poly(MEO_2_MA) Hydrogels

**Figure 4:**
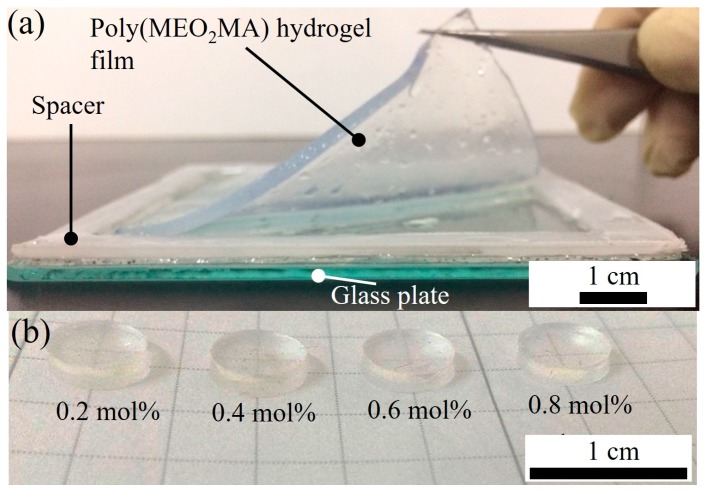
(**a**) Photo images of the fabricated poly(MEO_2_MA) hydrogel film by using the casting approach and (**b**) the disc-shape of poly(MEO_2_MA) hydrogel samples with four different molar feed ratios of cross-linker.

Poly(MEO_2_MA) hydrogel films with four different molar feed ratios of cross-linker were fabricated using casting approach, as presented in Figure 4a. Figure 4b shows the fabricated disc shape of the poly(MEO_2_MA) hydrogel samples with a diameter of 6 mm by using the hole-puncher. Each disc sample was rinsed and dried to achieve the weight constant and kept in the desiccator for further experiments.

### 3.2. Characterisations of Poly(MEO_2_MA) Hydrogel

The LCST and swelling/deswelling properties of the synthesised poly(MEO_2_MA) hydrogel with different molar feed ratios of cross-linker were characterised for fabricating the imiquimod-poly(MEO_2_MA) hydrogel dressing.

#### 3.2.1. Measurements of the LCST

**Figure 5:**
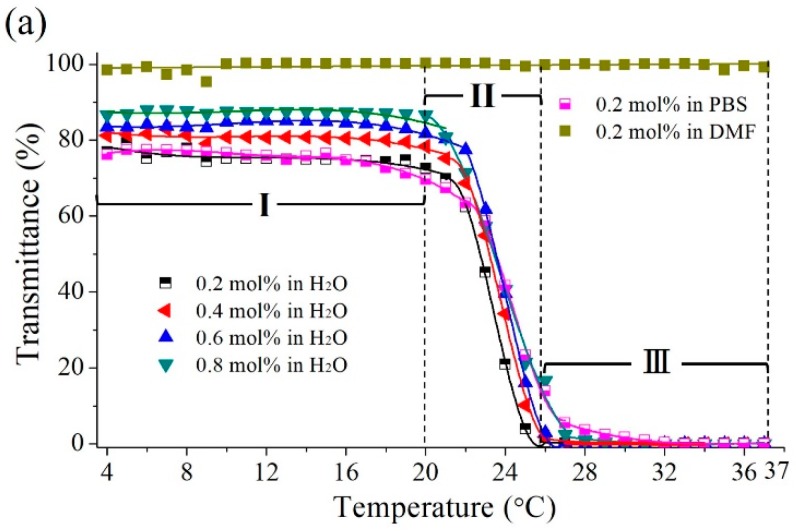

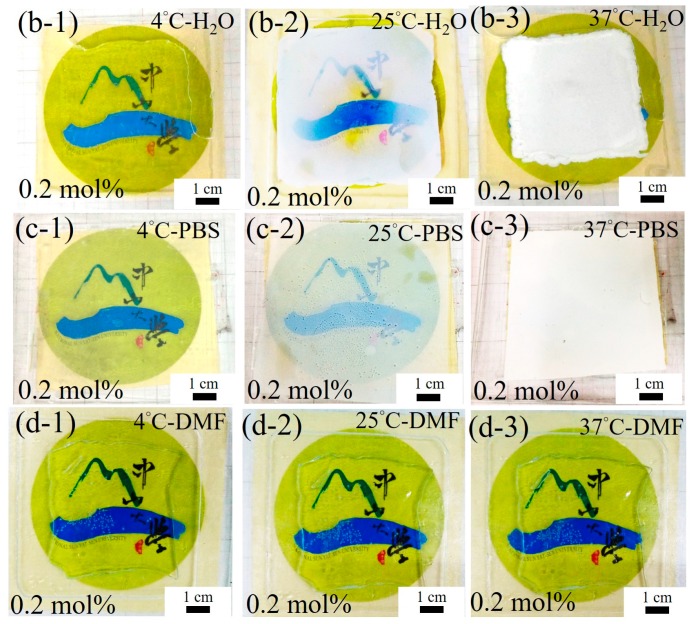
(**a**) Optical transmittance of poly(MEO_2_MA) hydrogel samples were measured as a function of temperature at 500 nm with the scan rate 2 °C min^−1^. (**b**–**d**) The transmittance changes of poly(MEO_2_MA) hydrogel pads were characterised in Milli-Q water, phosphate buffered saline (PBS), and dimethylformamid (DMF) solution under the temperatures of 4 °C, 25 °C, and 37 °C, respectively.

Figure 5a presents the temperature dependence of the optical transmittance of poly(MEO_2_MA) hydrogel, crosslinked with 0.2 mol %, 0.4 mol %, 0.6 mol %, and 0.8 mol % of DEGDMA samples, measured in Milli-Q water (control group), DMF, and PBS solutions, respectively. Images in Figure 5b–d present the transmittance variations corresponding to Figure 5a. The transmittance changes in Milli-Q water and PBS solution showed a similar trend, as can be seen from Figure 5a. In brief, the transmittance changes from 4 °C to 37 °C can be divided into 3 regions, which are Regions (I), (II), and (III). In Region (I), only 2.82% of transmittance was decreased from 4 °C to 20 °C. The poly(MEO_2_MA) hydrogel samples are retained in the transparent state, as shown in Figure 5(b-1,c-1). Then, the transmittance decreased dramatically in the temperature between 20 °C and 28 °C, as shown in Region (II). Within this temperature range, the hydrogel film changed to turbid, as presented in Figure 5(b-2,c-2). In this region, it clearly exhibits two transmittance boundaries, which are the transparent and the opaque state, caused by the phase behaviour changes of the polymer-solvent mixtures. The LCST values of four poly(MEO_2_MA) hydrogel samples measured in Milli-Q water were obviously observed in this region. The poly(MEO_2_MA) hydrogel, crosslinked with 0.2 mol % of DEGDMA samples in PBS solution, performed a similar transmittance variation, which indicates the LCST values were around 24 °C. As demonstrated in Figure 5(b-3,c-3), the samples turned to an opaque state in the Region (III) as the temperature increased to over 28 °C. Based on these observations, it is identified that the added amounts of the DEGDMA (cross-linker) do not affect the LCST values significantly.

DMF solvent performs a higher dissolving ability than Milli-Q water, hence it was selected as the drug carrier for imiquimod loading in this research. The poly(MEO_2_MA) hydrogel, crosslinked with 0.2 mol % of DEGDMA sample, was also tested by using the UV-Vis in the DMF solvent. No obvious transmittance changes (LCST) occurred between 4 °C and 37 °C, as shown in Figure 5a. It is verified by the photo images in Figure 5d. The transmittance states of poly(MEO_2_MA) hydrogel remained transparent from 4 °C to 37 °C.

#### 3.2.2. Swelling Profiles of the Poly(MEO_2_MA) Hydrogels

**Figure 6:**
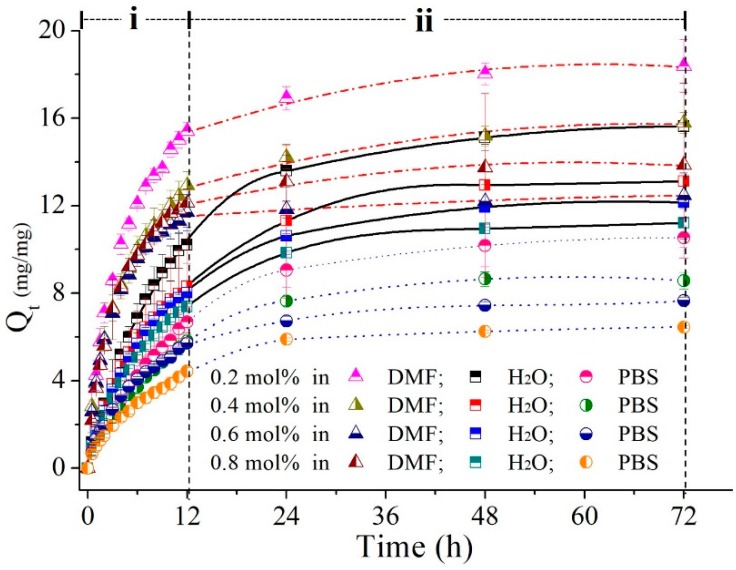
Dynamic swelling profiles of poly(MEO_2_MA) hydrogel samples measured in DMF, Milli-Q water, and PBS at constant temperature of 4 °C for 72 h, respectively.

The swelling ratios of poly(MEO_2_MA) hydrogel samples as a function of time were measured in Milli-Q water DMF and PBS solutions at 4 °C for 72 h, and the result was presented in Figure 6. The temperature of three solutions, which was 4 °C, was lower than the LCST of poly(MEO_2_MA) hydrogel, so that samples naturally turned to the swelling state, as presented in Figure 6. All samples performed the similar swelling trend in the 3 different solutions. The swelling ratios can be divided into two sections, which are the time interval between 0 and 12 h (i) and the second section from 12 to 72 h (ii). In the first period (i), the swelling ratios increased rapidly and then gradually increased in the second time-interval (ii). For example, the swelling ratio of poly(MEO_2_MA) hydrogel, crosslinked with 0.2 mol % of DEGDMA sample in DMF solution, had approximate 16-fold increase from the initial dry state in the first 12 h. Then, only 2-fold of the swelling ratio was increased from 12 h to 72 h. Moreover, the swelling ratio of the poly(MEO_2_MA) hydrogel, crosslinked with 0.2 mol % of DEGDMA, was 1.4-fold higher than the poly(MEO_2_MA) hydrogel, crosslinked with 0.8 mol % of DEGDMA sample. By comparing the swelling ratios measured in DMF solution, the lower swelling ratio in Milli-Q water and PBS was observed. The swelling ratio of the poly(MEO_2_MA) hydrogel, crosslinked with 0.2 mol % of DEGDMA sample operated in DMF, was around 18-fold increase. The results show that the added amounts of the DEGDMA cross-linker can affect the swelling ratio of the poly(MEO_2_MA) hydrogel samples, i.e., the lower the cross-linker amount that is added, the higher the swelling capability that is observed.

**Figure 7:**
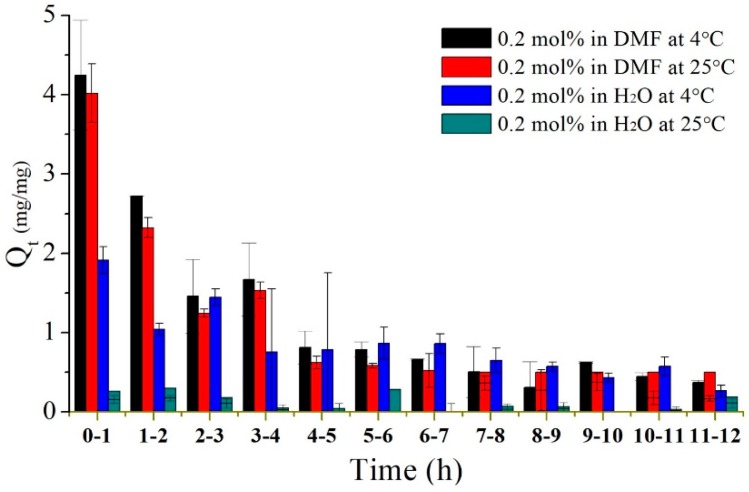
The swelling ratios of poly(MEO_2_MA) hydrogel, crosslinked with 0.2 mol % of DEGDMA samples, within 12 h in Milli-Q water and DMF solution.

The swelling response in the first 12 h, as indicated in Region (i) of Figure 6, was rapid and large. Therefore, swelling experiments were repeated to test the poly(MEO_2_MA) hydrogel, crosslinked with 0.2 mol % of DEGDMA in DMF solution and Milli-Q water at 4 °C and 25 °C, to investigate the swelling behaviours in detail. Figure 7 presents the swelling ratios of each hour during the first 12 h. There was no significant difference between the swelling ratios characterised at 4 °C and 25 °C in DMF solution. However, the measurements operated at 4 °C and 25 °C in Milli-Q water show obvious swelling responses. The poly(MEO_2_MA) hydrogel sample shows double swelling ratio at 4 °C increase but there was also no swelling response that occurred at 25 °C. This observation matches to the characterised results in Figure 5(a,b-2). Surprisingly, the poly(MEO_2_MA) hydrogel samples in the DMF solution performed a distinct swelling ratio increase and quick absorption speed in the first 4 h. The swelling ratio approached 4 in the first hour. These results prove that the DMF solution is the appropriate candidate for imiquimod loading.

#### 3.2.3. Deswelling Profiles of the Poly(MEO_2_MA) Hydrogels

**Figure 8:**
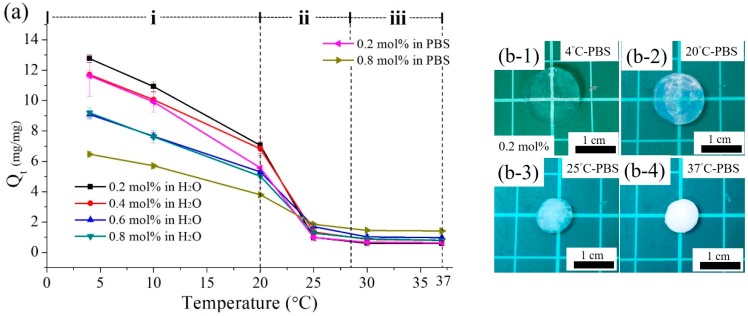
(**a**) Deswelling ratios of poly(MEO_2_MA) hydrogel samples were measured as a function of the temperature in Milli-Q water and PBS solution. (**b**) Optical images of the poly(MEO_2_MA) hydrogel, crosslinked with 0.2 mol % of DEGDMA, corresponding to the temperature changes between 4 °C and 37 °C.

Similar procedures were repeated to measure the deswelling ratios of the poly(MEO_2_MA) hydrogel samples as a function of temperature in Milli-Q water and PBS solution. The deswelling ratios that measured between 4 °C and 37 °C are presented in Figure 8a. Figure 8b demonstrates the images corresponding to the plot graphic. Six poly(MEO_2_MA) hydrogel samples show a decreasing trend in the deswelling ratios, and the deswelling ratios decrease as the temperature of solution increases. For example, the sample of the poly(MEO_2_MA) hydrogel, crosslinked with 0.2 mol % of DEGDMA, was characterised in Milli-Q solution. In Region (i), the deswelling ratio declined from approximate 13- to 7-fold in the temperature range from 4 °C to 20 °C. Then, the deswelling ratio dramatically decreased from 7.05- to 0.98-fold in Region (ii). Between 25 °C and 37 °C in Region (iii), the deswelling ratio was almost constant without any change. The samples of the poly(MEO_2_MA) hydrogel, crosslinked with 0.2 mol % and 0.8 mol % of DEGDMA, were measured from 4 °C to 37 °C in PBS solution. The deswelling ratios of two samples were approximately 12-fold and 6-fold during the temperature increase from 4 °C to 37 °C, respectively. As can be seen from Figure 8b, the volumes and transmittances of the poly(MEO_2_MA) hydrogel sample were affected by the changed solution temperatures.

**Figure 9:**
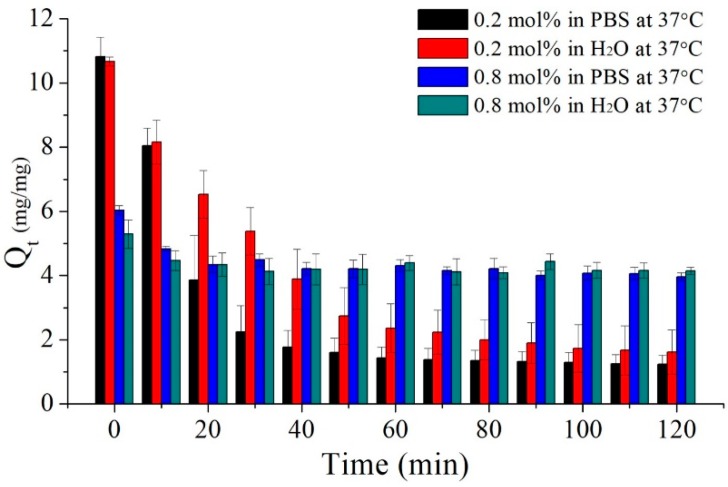
The bar chart presents the deswelling ratios of poly(MEO_2_MA) hydrogel, crosslinked with 0.2 mol % and 0.8 mol % of DEGDMA at 37 °C in PBS solution and Milli-Q water, respectively.

As the deswelling behaviour responds rapidly in the beginning of the deswelling procedure, the details of the deswelling ratios were measured by each every 10 minutes. Figure 9 presents the deswelling ratio of the poly(MEO_2_MA) hydrogel samples operated in PBS solution and Milli-Q water at 37 °C as a function of time. The deswelling experiment started from the immersion of the swollen samples of the poly(MEO_2_MA) hydrogel, crosslinked with 0.2 mol % and 0.8 mol % of DEGDMA in PBS solution and Milli-Q water at 4 °C for 72 h until the equilibrium state. As can be seen from Figure 9, the initial swelling ratios of the poly(MEO_2_MA) hydrogel, crosslinked with 0.2 mol % and 0.8 mol % of DEGDMA, were approximate 10.7-fold and 6.0-fold, respectively. The samples of the poly(MEO_2_MA) hydrogel, crosslinked with 0.2 mol % of DEGDMA, preformed dramatically the swelling ratio decrease from around 10.7-fold to 1.4-fold. Especially, there was around 70% of deswelling ratio decrease in the first 60 min in PBS solution. However, the deswelling response of the poly(MEO_2_MA) hydrogel, crosslinked with 0.8 mol % of DEGDMA, was mild, and there was only around 1.5-fold decrease from the equilibrant to the deswelling state in PBS solution.

### 3.3. Study of Imiquimod Loading and Releasing

The imiquimod-loaded amount and releasing profiles were characterised for the fabrication of imiquimod-poly(MEO_2_MA) hydrogel dressing. The analytical balance and UV-Vis were utilised to measure the amount of loaded and released imiquimod. The details on the amount of imiquimod loading and release profile are shown in the section below.

#### 3.3.1. Imiquimod Loading Profile

To understand the loading capability and imiquimod embedded amount of the poly(MEO_2_MA) hydrogel samples is an important step for evaluating the performance of the fabricated imiquimod-poly(MEO_2_MA) hydrogel dressing. Table 1 indicates the amount of loaded imiquimod in the poly(MEO_2_MA) hydrogel, crosslinked with 0.2 mol %, 0.4 mol %, 0.6 mol %, and 0.8 mol % of DEGDMA samples. It clearly shows that the lower the amount of the DEGDMA cross-linker added, the higher the imiquimod loaded. The following procedure and Equation (2) were utilised to estimate the amount of loaded imiquimod. For example, the imiquimod/DMF solution, composed of 15 mg of the imiquimod powder and 15 mL of DMF solution, was prepared and the whole solution weight was 14.2 g. After the immersion process, the weight of the absorbed solution was obtained by calculating the weight difference of the dry and swollen poly(MEO_2_MA) hydrogel sample. The amount of loaded imiquimod could be obtained by multiplying the weight of absorbed solution with the mass fraction of the solution, where the mass fraction of the solution, 0.001, was calculated by using Equation (2):

W_l_ = (*m_l_*/*m_c_*) (2)

where *m_l_* is the mass of dissolved imiquimod and *m_c_* is the mass of imiquimod/DMF solution.

The embedded amounts decreased from 27.4 μg/mm^3^ to 18.9 μg/mm^3^, as the added DEGDMA ratios increased from the 0.2 mol % to 0.8 mol %. Moreover, the loading process is based on the swelling behaviour and the loaded amount corresponds to the swelling ratios, as presented in Figure 6.

#### 3.3.2. Imiquimod-Release Profiles

**Figure 10:**
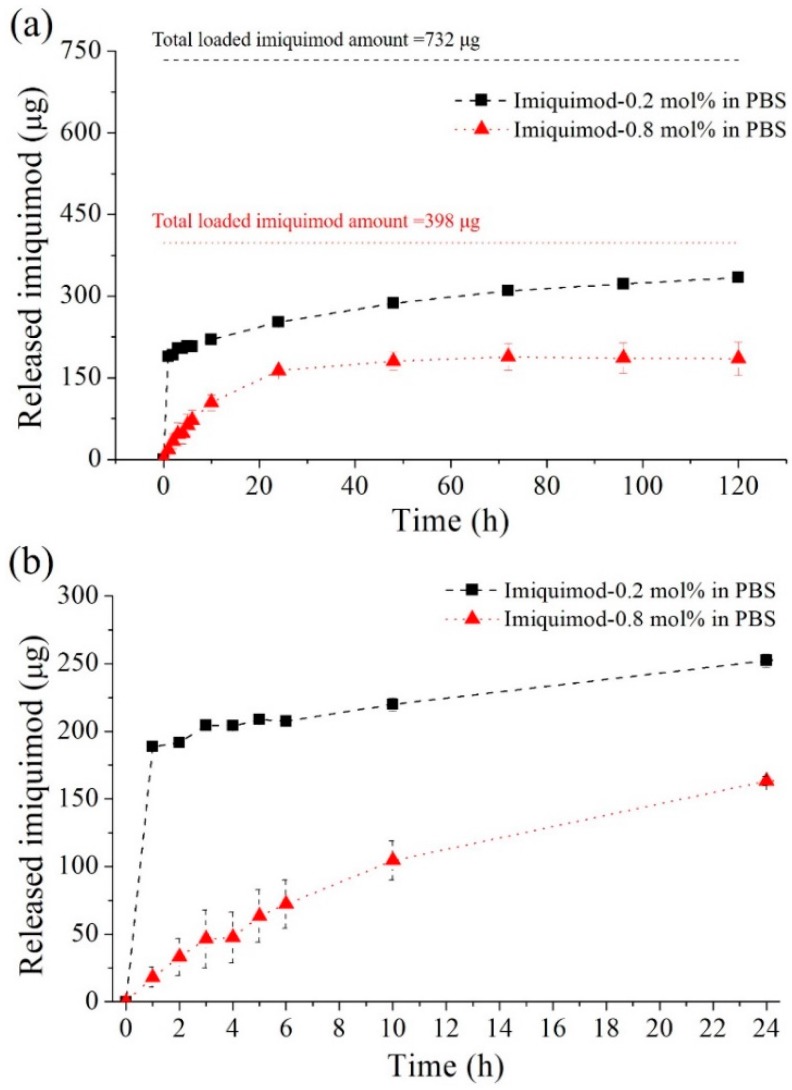
(**a**) The characterisation of imiquimod-release profiles from the samples of the imiquimod-poly(MEO_2_MA) hydrogel, crosslinked with 0.2 mol % and 0.8 mol % of DEGDMA in PBS solution at 37 °C. (**b**) The details of imiquimod-release profiles in the first 24 h.

The imiquimod-release amounts as a function of the time are shown in Figure 10a,b. The imiquimod-release profiles of the imiquimod-poly(MEO_2_MA), crosslinked with 0.2 mol % and 0.8 mol % of DEGDMA, were characterised in PBS solution at 37 °C for 120 h. The total loaded imiquimod amounts in the two imiquimod-poly(MEO_2_MA) hydrogel samples were 732 μg and 398 μg, which were obtained from 3 samples, respectively. As can be seen from Figure 10a, the released imiquimod amounts from two samples were less than half of total loaded imiquimod after 120 h. The released imiquimod amount of the imiquimod-poly(MEO_2_MA) crosslinked with 0.2 mol % of DEGDMA in PBS solution for 120 h was 344 μg, which was about 45% of total loaded imiquimod. The sample of the imiquimod-poly(MEO_2_MA), crosslinked with 0.8 mol % DEGDMA, shows that 46% of the imiquimod-release ratio and around 180 μg of imiquimod were released in 120 h. Interestingly, the imiquimod-poly(MEO_2_MA) crosslinked with 0.2 mol % of DEGDMA performs a more rapid release rate than the sample crosslinked with 0.8 mol % of DEGDMA in the first 60 min, as revealed in Figure 10b. The released amounts of imiquimod in the first hour from the samples crosslinked with 0.2 mol % and 0.8 mol % of DEGDMA were around 189 μg and 18 μg, respectively. By comparison of two different crosslinked ratios of samples, there was a distinct released amount difference in the first 60 min that matched to the deswelling behaviours demonstrated in Figure 9. The released amounts from the samples crosslinked with 0.2 mol % of DEGDMA increased mildly from 2 to 24 h, and the increased amount was approximately 35 μg. Between 24 and 120 h, the release amounts increased from about 82 to 344 μg. However, the imiquimod-poly(MEO_2_MA) crosslinked with 0.8 mol % of DEGDMA shows different imiquimod-release profile in the first 24 h. During the first 24 h, the released amounts increased mildly to approximately 163 μg. Then, there was also no increase in the imiquimod release amount.

### 3.4. Characterisation of Fabricated Imiquimod-Poly(MEO_2_MA) Hydrogel Pad

The CCK-8 assay and the ELISA were applied to measure the OD variations of the medium, which was used for culturing the keloid fibroblasts. The keloid fibroblasts were cultured separately in a dish (reference group), in the pure-poly(MEO_2_MA) hydrogel (control group), and in the imiquimod-poly(MEO_2_MA) hydrogel samples. The measured cell viability from the dish group was assumed to be 100% of the base line value. The measured OD value from the pure-poly(MEO_2_MA) hydrogel sample was divided by the base line value to obtain the cell viability of the pure-poly(MEO_2_MA) hydrogel samples as the control group. The same method was repeated to calculate the cell viability of the imiquimod-poly(MEO_2_MA) samples. The cell viability values of pure-poly(MEO_2_MA) hydrogel and imiquimod-poly(MEO_2_MA) hydrogel were compared to understand the effect of released imiquimod on the proliferation of the keloid fibroblasts.

**Figure 11:**
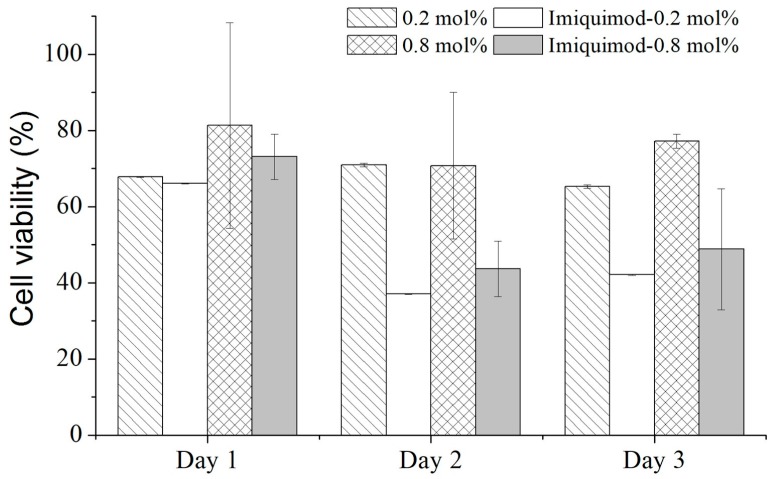
The cell viability was measured from keloid fibroblasts cultured on the polystyrene dish, pure-poly(MEO_2_MA) hydrogel, and imiquimod-poly(MEO_2_MA) hydrogel, crosslinked with 0.2 mol % and 0.8 mol % of DEGDMA by using Cell Counting Kit-8 (CCK-8) assay and enzyme-linked immunosorbent assay (ELISA).

Figure 11 presents the cell viability measured from the cultured keloid fibroblasts on the samples of the pure-poly(MEO_2_MA) and imiquimod-poly(MEO_2_MA) hydrogel, crosslinked with 0.2 mol % and 0.8 mol % of DEGDMA for three days. In the first day, there was no significant decrease in the cell viabilities between 4 samples, and the cell viabilities were around 70%. As can be seen from Figure 10a, the imiquimod-release amounts from the samples of the imiquimod-poly(MEO_2_MA) hydrogel, crosslinked with 0.2 mol % and 0.8 mol % of DEGDMA, were close to approximate 50% of total loaded imiquimod. From second days, the cell viabilities of two pure-poly(MEO_2_MA) hydrogel samples remained at around 70%. However, the samples of imiquimod-poly(MEO_2_MA) hydrogel, crosslinked with 0.2 mol % and 0.8 mol % of DEGDMA, performed obvious cell viability decreases that were affected by released imiquimod. Compared to the pure-poly(MEO_2_MA) hydrogel samples, there was around a 34% decrease of the cell viabilities on the second and third days. The research team presented similar results and the imiquimod was applied to inhibit the proliferation of the Langerhans cells. The cell viability decrease occurred after the second day, which demonstrated similar trend, as presented in Figure 11 [35]. Therefore, we believe that the developed and fabricated imiquimod-poly(MEO_2_MA) hydrogel pad had the ability to inhibit the proliferation of keloid fibroblasts.

## 4. Conclusions

In this research, we present the fabrication of imiquimod-poly(MEO_2_MA) hydrogel dressing for keloid therapy application. The fabrication procedures can be divided into three steps, including the synthesis of the poly(MEO_2_MA) hydrogel, the hydrogel film casting, and the imiquimod loading. The poly(MEO_2_MA) hydrogel, crosslinked with 0.2 mol %, 0.4 mol %, 0.6 mol %, and 0.8 mol % of DEGDMA, were synthesised separately and were shaped to have a membrane of 1 mm thickness. The basic properties of poly(MEO_2_MA) hydrogel films, such as the LCST, transmittance, and swelling/deswelling ratios, were measured for use in fabricating the hydrogel pad. The LCST of four poly(MEO_2_MA) hydrogels were measured at approximately 28 °C, which clearly indicates the turning point of swelling and deswelling behaviours. The swelling ratio of the poly(MEO_2_MA) hydrogel, crosslinked with 0.2 mol % of DEGDMA, was 1.4-fold bigger than the sample crosslinked with 0.8 mol % of DEGDMA at 4 °C. 

In drug loading process, the DMF solution was applied to load the imiquimod to the samples of the poly(MEO_2_MA) hydrogel, crosslinked with 0.2 mol % and 0.8 mol % of DEGDMA at 25 °C for 12 h. The net loaded amounts of imiquimod in two different types of the samples were approximately 27.4 μg and 14.1 μg per 1 mm^3^ of the hydrogel. In order to understand the deswelling property, two different types of the samples were measured in a PBS solution and the deswelling ratios of the two samples were approximately 12-fold and 6-fold during the temperature increase from 4 °C to 37 °C. The imiquimod-release profiles of the two groups of the samples were also characterised in PBS solution at 37 °C, and the released imiquimod amounts from two samples were less than half of the total loaded imiquimod after 120 h. The released imiquimod amounts were about 45% and 46% of the total loaded imiquimod.

The experiments of the imiquimod release and cell viabilities were applied to evaluate the performance of the fabricated imiquimod-poly(MEO_2_MA) hydrogel dressing. The CCK-8 assay was utilised to analyse the cell viabilities of keloid fibroblasts cultured on imiquimod-poly(MEO_2_MA) pads. There was an approximate 34% decrease in the cell viabilities between the samples of the pure-poly(MEO_2_MA) hydrogel and the imiquimod-poly(MEO_2_MA) hydrogel, crosslinked with 0.2 mol % and 0.8 mol % of DEGDMA after 2 days. It is verified that the proliferation of keloid fibroblasts could be inhibited by the released imiquimod. Therefore, the imiquimod-poly(MEO_2_MA) hydrogel pad has the potential to be applied to keloid therapy in clinical applications.

## Figures and Tables

**Table 1:** The loaded amount of imiquimod corresponding to the poly(MEO_2_MA) hydrogel samples with four different molar feed ratios of cross-linker.

Samples	0.2 mol %	0.4 mol %	0.6 mol %	0.8 mol %
Imiquimod (μg/mm^3^)	27.4 ± 0.55	21.7 ± 3.95	19.2 ± 5.44	14.1 ± 2.49
